# Endograft Collapse After Endovascular Aortic Aneurysm Repair With Acute Aortic Occlusion: A Case Report

**DOI:** 10.7759/cureus.101411

**Published:** 2026-01-13

**Authors:** Mutsunori Kitahara, Takuya Higuchi, Taro Nakazato, Tatsuya Ozaki

**Affiliations:** 1 Department of Cardiovascular Surgery, Kansai Rosai Hospital, Amagasaki, JPN

**Keywords:** aortic occlusion, complication, endograft collapse, endovascular reintervention, evar

## Abstract

We present the case of a rare complication of endovascular aortic aneurysm repair (EVAR), which is acute aortic occlusion due to proximal endograft collapse after EVAR. A 72-year-old man underwent EVAR and right internal iliac artery occlusion for aneurysms of the right common and right internal iliac arteries. One month after EVAR, the patient presented to the emergency room with an acute onset of bilateral lower-extremity pain and paraplegia. CT angiography revealed collapse of the proximal endograft and thrombosis of both iliac endografts. The patient underwent bilateral over-the-wire Fogarty thrombectomy and aortic cuff placement. Lower-extremity perfusion was restored upon completion of the procedure. The patient regained motor function and was discharged on postoperative day 6.

## Introduction

As endovascular aortic aneurysm repair (EVAR) for abdominal aortic aneurysms and iliac artery aneurysms becomes more widely used, reports of subsequent complications have increased [1]. Complications of EVAR are diverse and range widely in severity. Proximal endograft collapse in the abdominal aorta is an extremely rare and limb-threatening complication that has been reported with various treatment approaches and outcomes [2]. We report a case of acute aortic occlusion due to proximal endograft collapse that occurred despite adherence to the device’s instructions for use. The technique of endovascular treatment of acute aortic occlusion due to proximal endograft collapse is described.

## Case presentation

**Figure 1:**
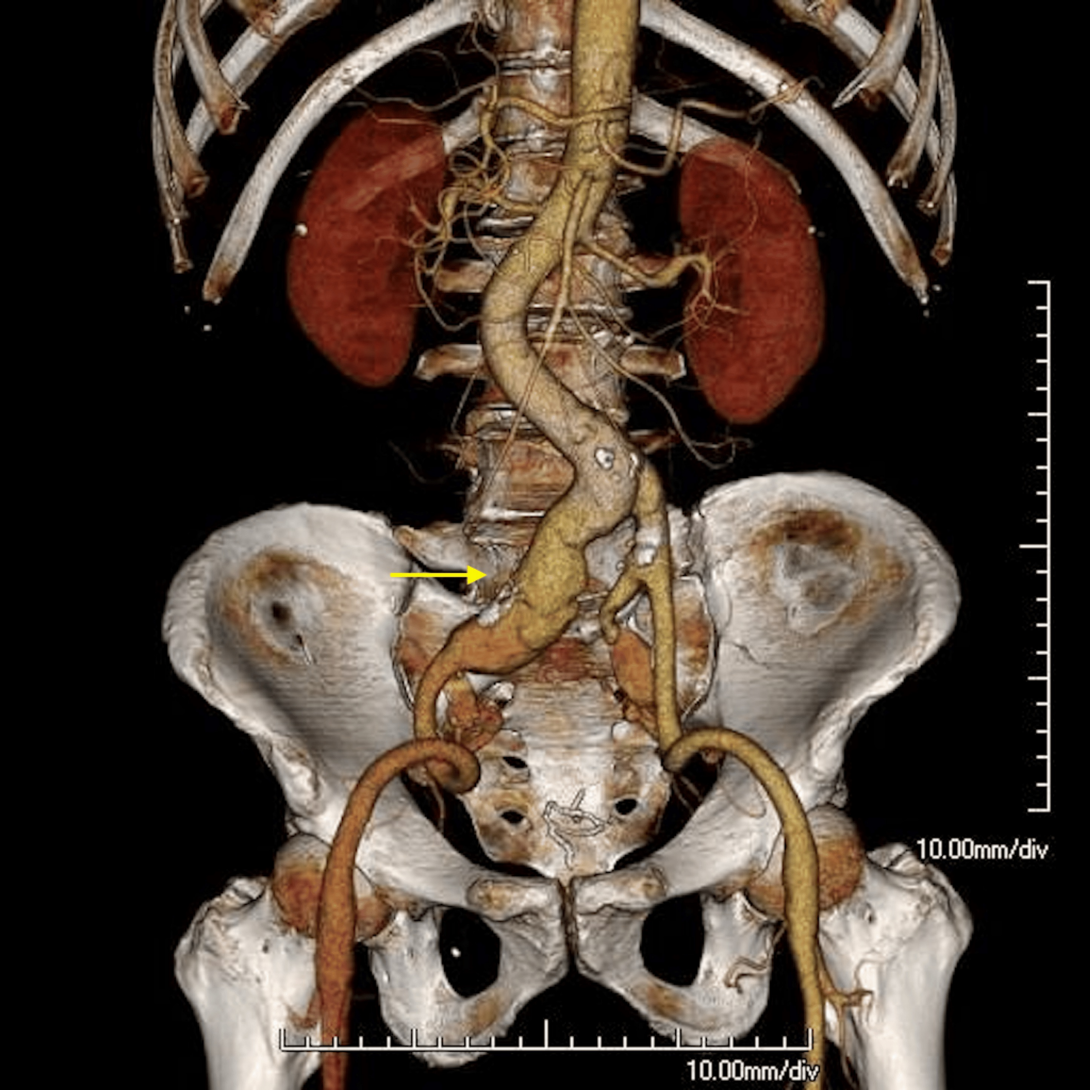
Volume-rendered reconstruction of CTA before EVAR The arrow indicates a right common iliac artery aneurysm measuring 35 mm. CTA, CT angiography; EVAR, endovascular aortic aneurysm repair

A 72-year-old man with a history of robot-assisted low anterior resection for rectal cancer presented with a right common iliac artery aneurysm measuring 35 mm, a right internal iliac artery aneurysm measuring 25 mm, and a left internal iliac artery aneurysm measuring 26 mm in diameter (Figure 1). The patient was scheduled to undergo EVAR because of his recent surgery for rectal cancer. It was decided that the left internal iliac artery aneurysm would be treated in two stages. The patient underwent right internal iliac artery occlusion, as preservation of right internal iliac artery flow was considered technically difficult.

A 5 Fr Destination guiding sheath (Terumo, Tokyo, Japan) was inserted and advanced into the right internal iliac artery. A 12 mm Amplatzer Vascular Plug II (Abbott Medical, Santa Clara, California, USA) was deployed through the sheath, and the right internal iliac artery was embolized. Metallic coils were used to assist in plug embolization.

**Figure 2:**
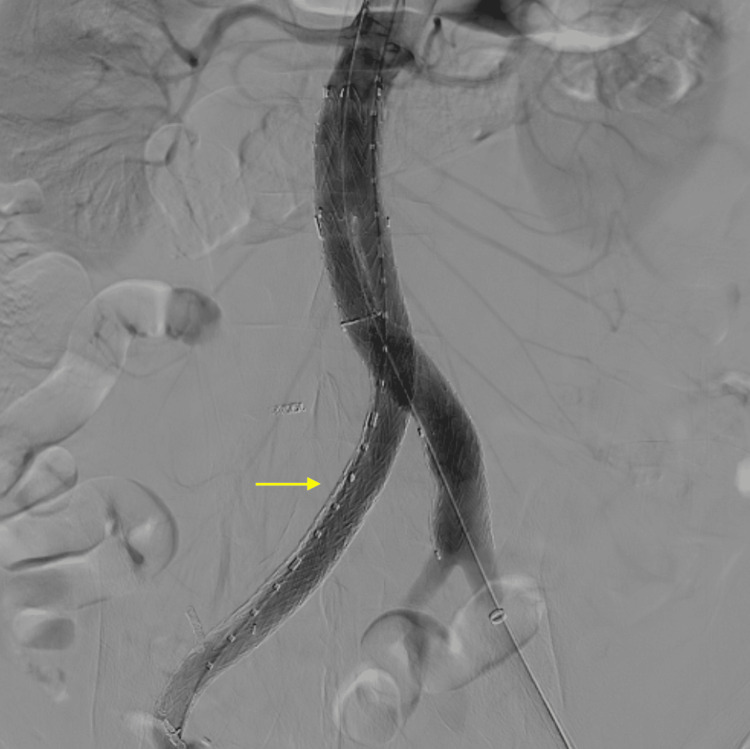
Completion aortography Completion aortography shows successful exclusion of the aneurysms and good graft apposition. The arrow indicates no endoleak.

At the same time, the patient underwent EVAR using a GORE Excluder conformable device (W. L. Gore and Associates, Flagstaff, Arizona, USA). The diameter of the healthy infrarenal aorta was 19-21 mm, and neck angulation was 42 degrees. A 23 mm diameter main body was used. A 20 mm leg was implanted into the left common iliac artery, which had a diameter of 15-19 mm, and a 12 mm leg was implanted into the right external iliac artery, which had a diameter of 10 mm. Post-deployment touch-up ballooning was performed at the landing zones and overlapping sites. Completion aortography revealed successful exclusion of the aneurysms and good graft apposition (Figure 2).

**Figure 3:**
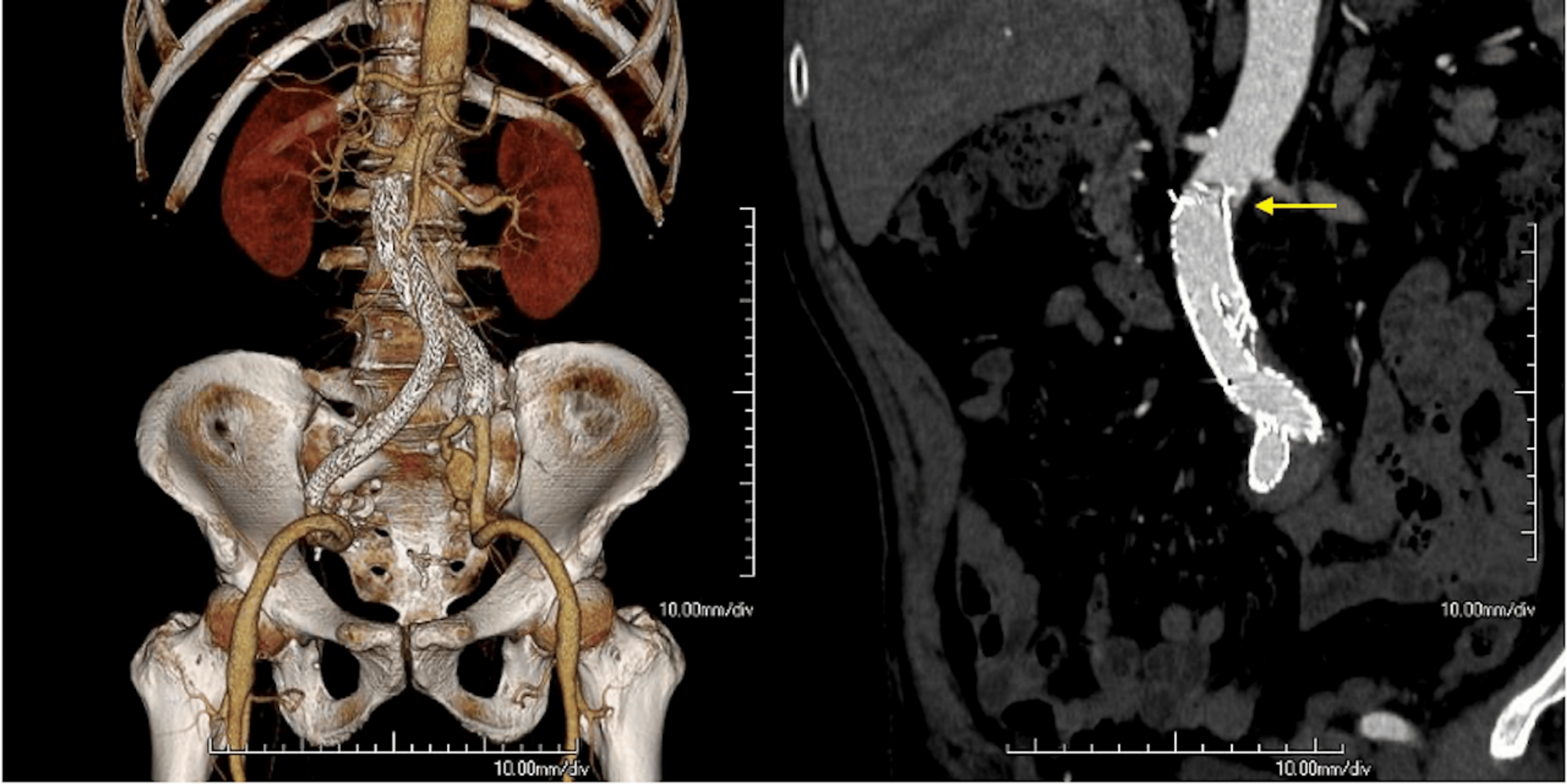
Volume-rendered reconstruction and coronal section of CTA six days after EVAR The arrow indicates a small bird-beak appearance, which was caused by slightly lower deployment of the endograft. CTA, CT angiography; EVAR, endovascular aortic aneurysm repair

The patient was discharged on postoperative day 3. CT angiography (CTA) performed six days after the procedure revealed no endoleak of the right common iliac artery aneurysm or right internal iliac artery aneurysm and a small bird-beak configuration of the proximal endograft (Figure 3). No additional procedures were performed.

**Figure 4:**
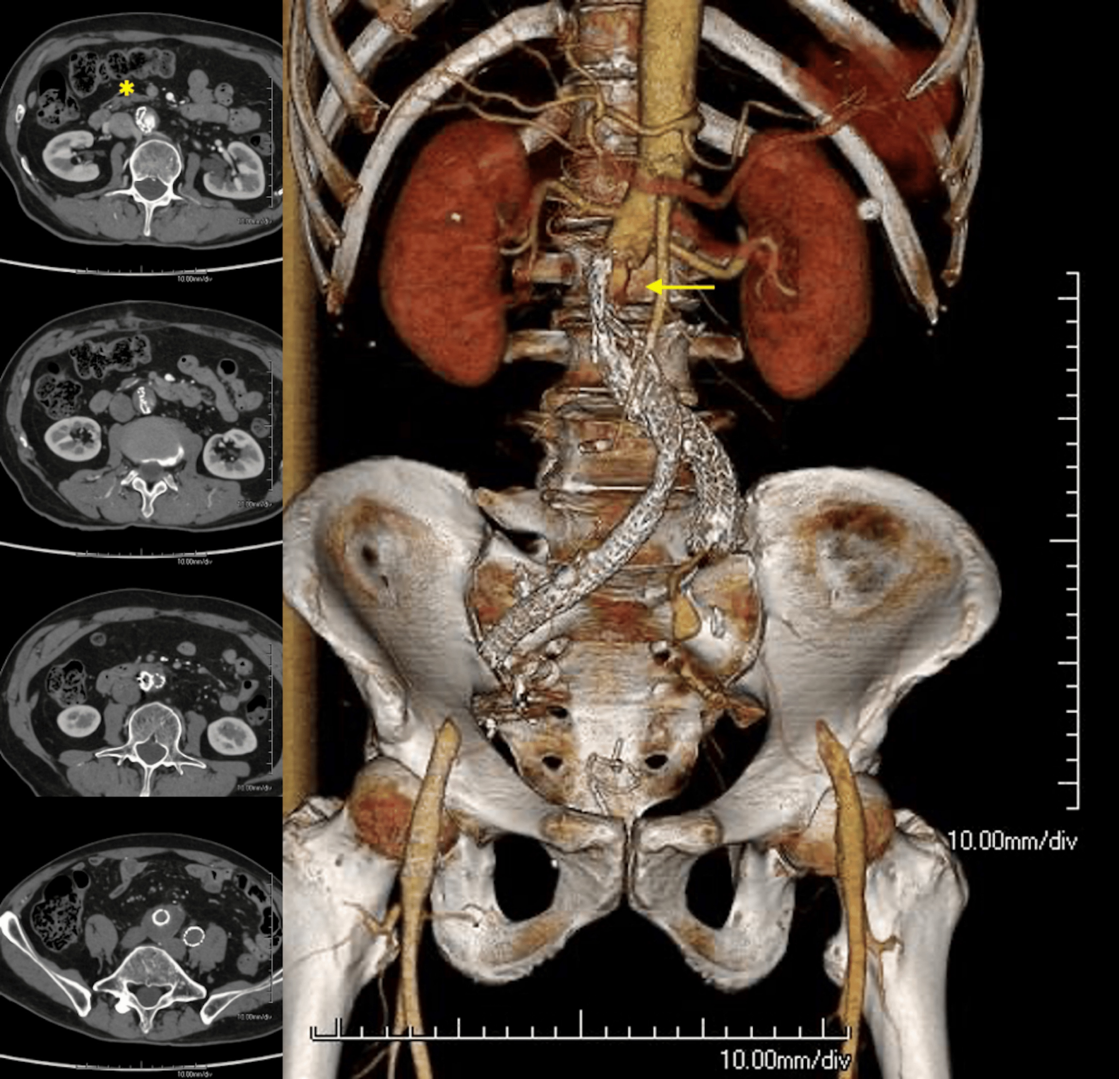
Axial sections and volume-rendered reconstruction of CTA one month after EVAR The arrow indicates the collapse of the proximal endograft, which caused acute aortic occlusion and thrombosis of the bilateral common iliac limbs. The axial image demonstrates the collapsed main body (^*^). CTA, CT angiography; EVAR, endovascular aortic aneurysm repair

One month after the procedure, the patient presented to the emergency department with acute-onset bilateral lower-extremity pain and paraplegia. Physical examination revealed the absence of femoral and pedal pulses, and the lower extremities were cool to the touch. CTA revealed the collapse of the proximal endograft with thrombosis of the bilateral common iliac limbs (Figure 4).

Endovascular treatment was considered feasible because the initial EVAR had been successfully performed in accordance with the device’s instructions for use. The patient was subsequently transferred to the operating room. Under general anesthesia, bilateral femoral artery cutdown and over-the-wire Fogarty thrombectomy were performed. A 0.035-inch Radifocus guidewire (Terumo) was advanced into the descending thoracic aorta. The wire was then exchanged for an Amplatz extra-stiff guidewire (Cook Medical Japan G.K., Tokyo, Japan) over a JR40 Goodtec angiographic catheter (NIPRO, Osaka, Japan). The proximal endograft re-expanded spontaneously. Once the inflow was restored, a 26 mm aortic cuff (W. L. Gore & Associates) was placed proximal to the preexisting endograft to provide additional radial force and achieve better apposition. Lower-extremity perfusion was restored upon completion of the procedure, and the patient regained motor function.

**Figure 5:**
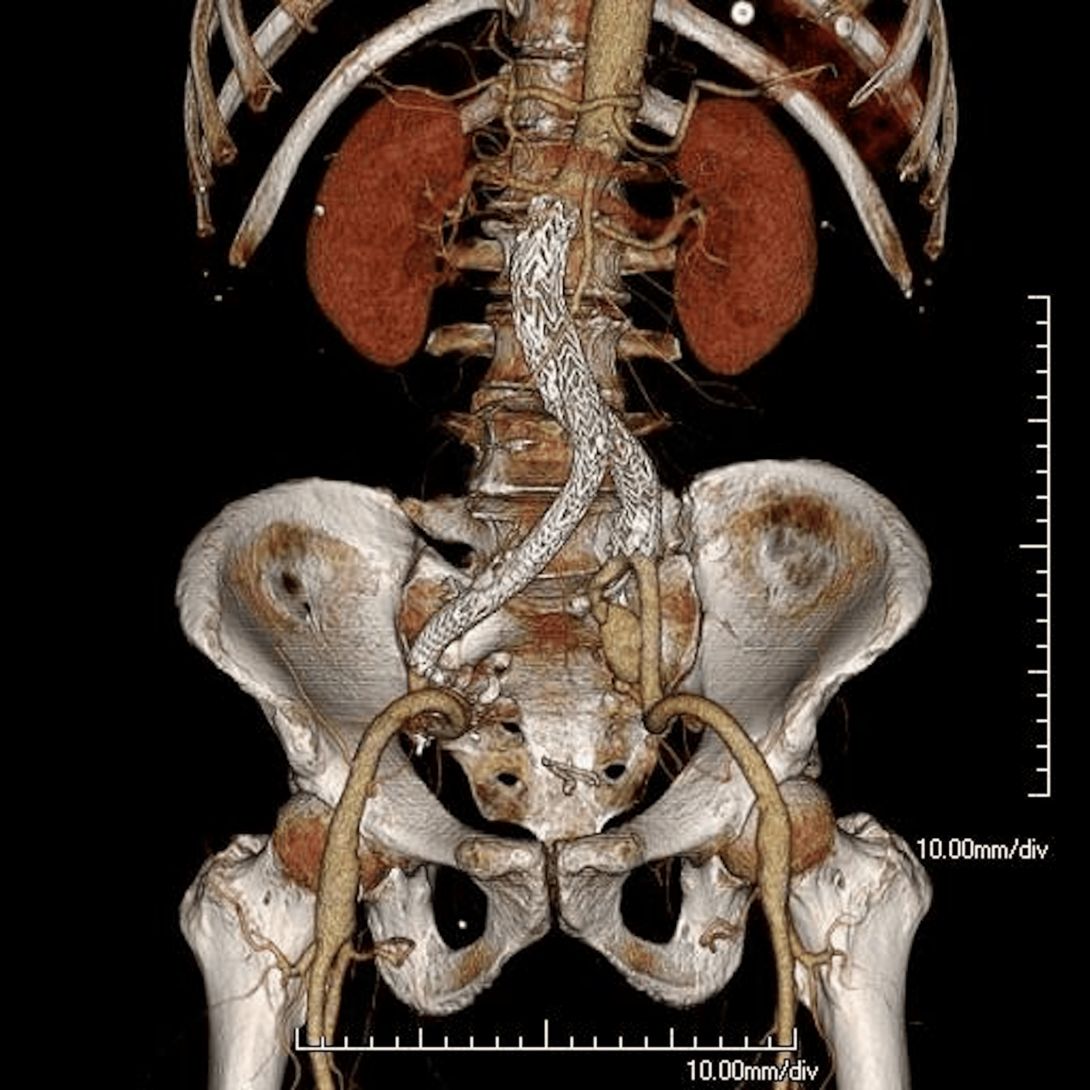
Volume-rendered reconstruction of postoperative CTA CTA demonstrates full expansion of the proximal endograft five days after the second procedure. CTA, CT angiography

Follow-up CTA performed five days after the second procedure revealed full expansion of the proximal endograft and restored patency of the endograft (Figure 5). The patient was discharged on postoperative day 6.

## Discussion

Case reports of endograft collapse after EVAR are rare, particularly those presenting with acute aortic occlusion. Rathore et al. reviewed cases of proximal endograft collapse after EVAR and reported that the causes of aortic occlusion differed between the acute phase (occurring within 30 days) and the late phase. The main causes of aortic occlusion in the late phase include aortic dissection, graft migration, and endograft kinking. In contrast, the main causes of aortic occlusion in the acute phase have been reported to be related to aortic anatomic configuration [2]. In the present case, CTA confirmed the absence of aortic dissection or graft migration. Therefore, other factors were responsible for the proximal endograft collapse. These factors can be categorized as anatomical, procedural, and device-related, each potentially contributing to the observed event.

Excessive endograft oversizing is a potential risk factor for acute proximal endograft collapse [3]. In this case, graft oversizing was 11.1% (23 mm/20.7 mm), which was within the recommended instructions for use. Therefore, excessive oversizing was unlikely to be the primary cause. Angulation of the proximal neck of an aneurysm is another recognized risk factor for acute proximal endograft collapse [3]. In this case, the angulation of the infrarenal aorta was 42 degrees. The main body was positioned at the proximal neck curvature. After deployment, the aorta tended to straighten due to endograft correction. The force of the aorta returning to its original curvature might have compromised proximal endograft fixation. However, this degree of proximal neck angulation alone is not a contraindication to EVAR.

The main body was inserted via the right femoral artery during the initial procedure, resulting in a non-C-curve deployment. Slightly lower deployment of the endograft led to a bird-beak configuration, in which a portion of the proximal endograft protruded intraluminally after the procedure. A technical error was also identified in the performance of completion angiography without removal of the stiff guidewire, which led to overlooking the bird-beak configuration [4]. While these procedural factors may appear benign in most patients, they can contribute to endograft collapse when combined with other adverse factors. Importantly, the procedure in this case was performed within the recommended device instructions, reinforcing the notion that the causes of endograft collapse are likely multifactorial rather than attributable to a single technical error.

Device design characteristics are also important contributors. The use of a suprarenal stent, which is a bare stent on the proximal side of the endograft, allows the proximal portion of the endograft to follow the curvature of the aorta and may prevent a bird-beak configuration [5]. Rathore et al. reported four cases of endograft collapse after EVAR in the acute phase, all of which involved the use of a non-suprarenal stent device [2]. Caution should be exercised when using a non-suprarenal stent device in cases in which the proximal neck of the aneurysm exhibits even slight angulation. In certain cases, use of a suprarenal stent device may be preferable, depending on the individual aortic anatomy.

Proximal endograft collapse requires emergency treatment when patients present with signs of acute aortic occlusion; however, consensus regarding optimal treatment strategies has not yet been established [2]. Endovascular [3,6], hybrid [7,8], and open surgical repair [9,10] approaches have been successfully performed. Treatment options depend on the underlying etiology and should aim to restore blood flow as rapidly as possible. In this case, endovascular treatment was selected. If endovascular treatment had been unsuccessful, surgical repair with axillofemoral bypass would have been indicated. Relining with an aortic cuff was chosen to provide greater radial force than the collapsed endograft. In addition, fixation of the new endograft allowed extension of aortic coverage sufficiently proximal to the angulation, thereby providing stable fixation to the aortic wall.

## Conclusions

We describe a case of endograft collapse resulting in acute aortic occlusion, a rare complication of EVAR. This case highlights that endograft collapse may occur due to a complex interplay of anatomical, procedural, and device-related factors. Such complications can arise from multifactorial causes, even when the device’s instructions for use are followed. Early postoperative CTA in our patient might have provided a clue to impending graft collapse. Prompt detection and timely reintervention are essential to minimize the risk of limb-threatening complications.
